# Wikipedia, Google Trends and Diet: Assessment of Temporal Trends in the Internet Users’ Searches in Italy before and during COVID-19 Pandemic

**DOI:** 10.3390/nu13113683

**Published:** 2021-10-20

**Authors:** Daniele Nucci, Omar Enzo Santangelo, Mariateresa Nardi, Sandro Provenzano, Vincenza Gianfredi

**Affiliations:** 1 Nutritional Support Unit, Veneto Institute of Oncology IOV-IRCCS, Via Gattamelata 64, 35128 Padua, Italy; daniele.nucci@iov.veneto.it (D.N.); mariateresa.nardi@iov.veneto.it (M.N.); 2 Regional Health Care and Social Agency of Lodi, ASST Lodi, Piazza Ospitale, 10, 26900 Lodi, Italy; omarenzosantangelo@hotmail.it; 3 Local Health Unit of Trapani, ASP Trapani, Via G. Mazzini, 1, 91100 Trapani, Italy; provenzanosandro@hotmail.it; 4 School of Medicine, Vita-Salute San Raffaele University, Via Olgettina 58, 20132 Milan, Italy; 5 CAPHRI Care and Public Health Research Institute, Maastricht University, Minderbroedersberg 4-6, 6211 LK Maastricht, The Netherlands

**Keywords:** big data, Wikipedia, diet, Wikitrends, Google Trends

## Abstract

We obtained data from Google Trends and Wikipedia in order to assess whether an analysis of Internet searches could provide information on the Internet users’ behaviour/interest in diets. Differences in seasonality, year and before/during COVID-19 pandemic were assessed. From Wikipedia, we extracted the number of times a page is viewed by users, aggregated on monthly and seasonal bases. We also used Google Trends to evaluate the frequency of the users’ web searches. The Mediterranean diet was the most frequently (33.9%), followed by the pescatarian diet (9.0%). Statistically, significant seasonal differences were found for the Mediterranean, vegetarian, Atkins, Scarsdale, and zone diets and pescetarianism. The most commonly searched diet and consequent diet-related queries on Google resulted to be: Dukan diet, Dukan and weight loss. Ketogenic, FODMAP and intermittent fasting diets were statistically more frequently searched during the pandemic compared with before. Our data show a different trend of searches based on the seasonality, year and the pandemic. These data could be useful for scientists, practitioners and policy makers because they can inform educational campaigns via the Internet, especially in periods when the population is more receptive.

## 1. Introduction

Nowadays, an increasing number of people pay attention to their health [1]. Thanks to the impressive spread of the Internet and its easy access to a large amount of information (not even correct), the web is becoming one of the most trusted sources of information, even about health [2]. This is also true considering health aspects related to diet [3,4,5]. In the current digital era, an increasing number of original data sources and high amounts of data, called “Big Data”, are even more available for several uses, including health-research activities [6,7,8]. According to De Mauro et al., “[Big Data] represent resources/assets of an informative nature characterised by such a high volume, speed and variety as to require technology and analytical methods specific for its transformation into value” [9]. Big Data are characterised by the so-called 4Vs, volume, variety, velocity, and value [10], which refer to the amount of generated data, the different types of data, the rapidity of data transfer, and the value that can be obtained by analysing these amounts of data, respectively [10]. However, recently, two additional qualities have been ascribed to Big Data: variability and veracity. Variability represents the consistency of the data over time, whereas veracity refers to the accuracy, credibility, truthfulness of the data. Big Data include a novel data stream, which is defined by Althouse et al. as “those data stream whose content is initiated directly by the users (patients) themselves” [11]. Among them, Wikitrends is a promising new analytics framework for Wikipedia, offering the number of visualisations of Wikipedia pages [12]. Wikipedia is a free, non-profit online encyclopaedia, created and edited by volunteers from around the world. Wikipedia uses the power of the online community to create and edit encyclopaedia-like articles which are then available for free. Currently operating in 303 languages, Wikipedia has around 1.5 million articles available in Italian [13]. With a wealth of detailed information on an almost unlimited range of topics, Wikipedia is a platform that could potentially be useful for scientific research in many different areas [6,7,8,14,15].

According to a previous publication, Internet users frequently seek diet-related information in order to find healthy recipes, search for healthy diet recommendations and motivational information to change their diets, and lastly, lose weight most frequently in the preparation for holidays [16]. Moreover, it should be considered that diet is one of the health-related factors more influenced by trend. This is due to the progress of science but also marketing. Indeed, the popularity of diet may be highly influenced not (only) due to scientifical soundness, but due to efficient marketing. In this respect, some examples are the Atkins diet, intermittent fasting, Weight Watchers, the gluten-free diet, detox diet, alkaline diet, Palaeolithic diet, vegan diet, and macrobiotic diet [17,18]. Moreover, other external factors may have an influence on diet. Just as an example, the several containment measures adopted to contain the COVID-19 pandemic largely impacted many aspects of daily life and humans’ behaviours, for instance: mental health [14], physical activity [19], social interaction [20], and also diet [21,22]. However, all the previous studies used information collected by means of a survey. Despite the undoubtable advantages, such as being cheap, easy and a non-invasive method of assessment, the use of surveys is affected by some biases such as social-desirability bias and recall bias, especially if related to diet. Moreover, selection bias could represent another study error that impacts on the representativeness of the sample and, therefore, on the generalisability of results. Additionally, if the majority of previous studies focused on food intake to estimate adherence to a particular diet, others assessed food behaviour or preferences. On the contrary, none of the previous research used Wikipedia or Google to assess the internet users’ interest in diet. 

Considering the lack of knowledge, the vast use of the Internet for seeking dietary information, and the large amount of attention paid to diet, we assumed that assessing Wikipedia users, by analysing Wikitrends, can give more insights on Internet users’ search behaviours regarding diet over time, understanding which diets are more fashionable among the general population and whether there is a seasonality in the searching activities. Lastly, in the current analysis, differences in search volume on diet before and during the COVID-19 pandemic were also assessed.

## 2. Materials and Methods

The data for this study were collected from Wikipedia [23], the most frequently used encyclopaedia portal. From Wikipedia, we extracted the number of times a specific page is viewed by users; the data were extracted as daily data from July 2015 (from inception) to January 2021 (the last available data at the time of the analysis). Therefore, data were aggregated on a monthly and then seasonal basis (Spring: March–April–May; Summer: June–July–August; Autumn: September–October–November; Winter: December–January–February). The one-way ANOVA test was carried out to evaluate the differences between the seasonal averages. The searches for the pages in Italian were selected, and the diets considered were: Mediterranean, vegetarian (semi-vegetarian), ketogenic, Atkins, FODMAP, acid–base, vegan, blood groups, palaeolithic, Scarsdale, Kousmine method, zone diet, intermittent fasting, pescetarianism, fruitarianism, raw food, macrobiotics; the name of the Italian pages of the diets were: Mediterranea, Vegetariana (semivegertarianismo), Chetogenica, Atkins, FODMAP, Acido-base, Vegana (Vegetaliana), del gruppo sanguigno, Paleolita (Paleodieta), Scarsdale, Metodo Kousmine, dieta a zona (metodo alimentare a zona), digiuno intermittente, Pescetarianismo, Fruttarismo, Crudismo, Macrobiotica (defined in Supplementary Table S1). Moreover, Student’s t-test was used to assess differences between the number of times a specific diet page has been searched before and during the COVID-19 pandemic. The time period between July 2015 and February 2021 was defined as “before COVID-19 pandemic”, considering that, in Italy, the first autochthon case was detected on the 20 February 2021 [24].

Additionally, Google Trends, a big web-based open-source tool that assesses the frequency of web searches of populations, offering a comparison in trends stratified by location, time, category and search type, was used. In the current investigation, Google Trends was mined from inception (1 January 2004) up to 26 March 2021 (the last available data at the time of the analysis), searching for the word “diet” (in Italian “dieta”).

Distribution normality was tested by Shapiro–Wilk test. The statistical significance level for the analyses conducted was 0.05. The data were analysed using the STATA statistical software, version 16.0 (Crop LLC, College Station, TX, USA).

## 3. Results

**Figure 1:**
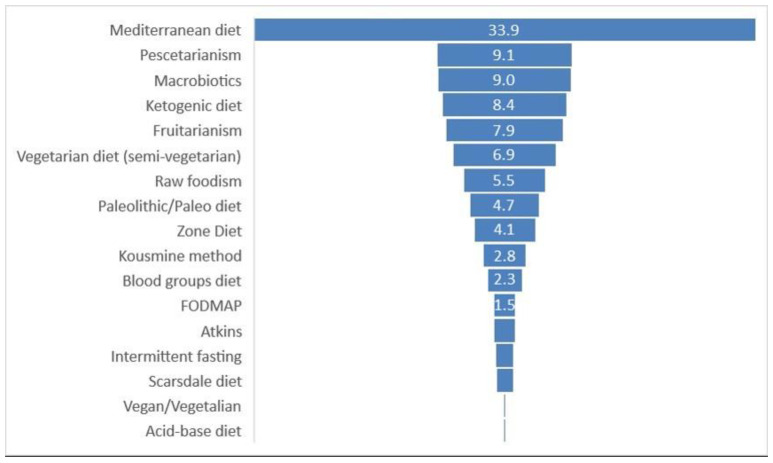
Percentages of the number of times a specific page was viewed by users out of the total number of times all pages were consulted.

**Figure 2:**
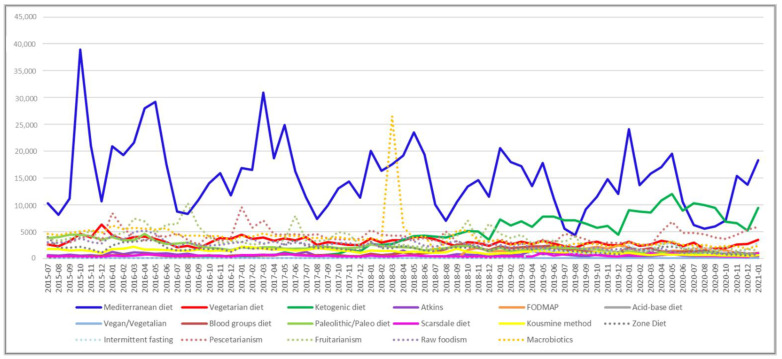
Wikipedia curves with reference to the number of page views for Mediterranean, vegetarian (semi-vegetarian), ketogenic, Atkins, FODMAP, acid–base, vegan, blood group, paleolithic, Scarsdale, Kousmine method, zone, intermittent fasting, pescetarianism, fruitarianism, raw foodism, and Macrobiotics diets.

As shown in Figure 1, the Mediterranean diet is the most frequently searched diet, with one user out of three searching for it. The second most frequently searched are the pescetarianism and Macrobiotics diets, followed by the ketogenic diet. On the contrary, the Atkins, intermittent fasting, Scarsdale, vegan/vegetalian and acid–base diets are less often searched with a frequency below 1.5%. Diet-related digital behaviour showed a seasonality throughout the study period with a peak during spring, considering the data obtained both from Wikitrends (Figure 2) and Google Trends (Supplementary Figure S1). Moreover, diet-related digital behaviours also showed a variability among the years (Supplementary Figure S2) and during the COVID-19 pandemic (Supplementary Table S1). Statistically significant seasonal differences were found for the Mediterranean (*p* < 0.001), vegetarian (*p* = 0.020), Atkins (*p* < 0.001), Scarsdale (*p* = 0.001), zone (*p* = 0.03) and pescetarianism (*p* = 0.04) diets, as reported in Table 1. During the COVID-19 pandemic, the ketogenic, FODMAP and Intermittent fasting diets were searched more compared with before the COVID-19 pandemic, in a statistically significant manner (Supplementary Table S1). On the contrary, vegetarian, vegan/vegetalian, Atkins, Kousmine, zone, fruitarianism, and raw foodism diets were significantly less searched during the COVID-19 pandemic compared to before (Supplementary Table S1). All the others did not show any statistically significant differences.

Table 1 also reports the results of the Shapiro–Wilk test. All the data are normally distributed since all the *p*-values obtained were >0.05.

Table 2 reports the first 25 most commonly searched and consequent diet-related queries. Dukan dieta, Dukan (Dukan diet, Dukan) and Dimagrire (weight loss) were the first commonly searched terms in Google. On the contrary, Detox/Dieta gravidanza/Dieta vegana (Detox/Diet in pregnancy/Vegan diet) and Dieta ipocolarica/dieta detox (low-calorie diet/detox diet) were the least searched terms.

## 4. Discussion

In this paper, Internet users’ search behaviours regarding diet over time, by analysing Wikitrends, have been investigated. This allowed us to identify which diets were more frequently searched and whether there is a seasonality in the searching activities. Based on our results, the Mediterranean diet, pescetarianism, macrobiotic and ketogenic diets were the top four more fashionable diets. Interestingly, the Mediterranean diet was characterised by the users’ consistent and constant interest throughout the study period, similar to pescetarianism, even if at a lower scale. On the contrary, the macrobiotic and ketogenic diets had a completely different pattern. Indeed, the macrobiotic diet showed an important spike in March 2018, whereas the ketogenic one showed an increasing interest starting from June 2019. Searching on the web for potential reasons, it has been found that, in Italy, the macrobiotic diet was mainly promoted by Mario Pianesi. He was an entrepreneur of the food sector, founder of the “Un Punto Macrobiotico”, a macrobiotic association, with many locations in Italy. According to what was reported by the media, it seems that the members of this association were required to follow a restrictive regimen, not only dietetic (usually the macrobiotic diet is a mainly vegetarian dietary pattern with a preference for organic, local and whole foods), but it seems that they were also obligated to avoid official medicine to treat diseases. For these reasons, he underwent legal proceedings. Investigations were conducted in March 2018, when many tabloids (also online) relaunched the news about Mario Pianesi and the macrobiotic association. Considering all the above-mentioned aspects, we hypothesised that the peak in the macrobiotic diet registered in March 2018 in the Wikipedia search volume could be related to this media event. For this reason, we assessed on Google trends the research trends of the word “Mario Pianesi” in Italy from 1 January 2004 to 26 March 2021. Even in this case, a peak in the research activity was found in March 2018 (Supplementary Figure S3a). Moreover, Google Trends also offers the possibility to assess the geographical distribution of the search volume. We noticed that the highest research volume was recorded in the Marche Region, where the police investigation mainly took place (Supplementary Figure S3b). 

As previously mentioned, the Mediterranean diet search volume is stably high overtime, despite the fact that it is the “oldest” diet among those assessed in this work. Indeed, the Mediterranean diet was first identified by Ancel Keys during the 1960s. Moreover, this is the diet with the highest number of high-quality studies that have revealed the strong association between the Mediterranean diet and a lower risk of several conditions such as, for instance, cardiovascular disease [25], several forms of cancer [26], mental disorders [27,28] and overall mortality [29]. Although the Mediterranean diet continues to remain the most frequently searched, emerging diets have been attracting the interest of the general population. Among them, the diet that has received increasing attention is the Ketogenic diet, which is not supported by the same amount/quality of evidence as the Mediterranean diet (Supplementary Figure S4 reports the number of articles for ketogenic, Mediterranean and macrobiotic diets in PubMed by year). In fact, the increasing success of the ketogenic diet is mainly ascribable to the weight loss effect observed; however, studies have failed to prove the beneficial effect of the ketogenic diet in treating obesity or diabetes [30]. Moreover, the ketogenic diet is the last issued low-carbohydrate diet, preceded by the palaeolithic and Atkins diets. This trend was also confirmed by our results, according to which the research volume regarding the palaeolithic and Atkins diets has decreased over time. 

Moreover, our results show statistically significant differences in search volume during seasons, showing a high amount of research during the spring. This is an interesting aspect that should be taken into account by experts in nutrition because it highlights the public’s need for information on diet, particularly in spring. This is indeed true considering that on the web a large amount of (dis)information is available to the general public who may not always have the appropriate knowledge to select good-quality (preferable scientific) contents and interpret them appropriately [31,32]. Further, our results, considering both the data obtained from Wikitrends and Google Trends, show which diets are more fashionable among the general public and consequently to which diet researchers should pay more attention to in order to improve the scientific dissemination on the web. Indeed, websites containing high-quality information on the types of diets and their potential health effects are fundamental in our society, where citizens (and even patients) like to be informed, or sometimes overinformed. In this context, the role of science communicators (in this case mainly represented by dieticians and nutritionists) should adequately cover and make the topic interesting for Internet users [33,34].

Lastly, in our analysis, differences in the visualisation of Wikipedia pages before and during the COVID-19 pandemic was conducted. Our data show that the internet users’ interest during the pandemic was mainly related to ketogenic, FODMAP and intermittent fasting diets. The increased trend for ketogenic and intermittent fasting diets could be explained because some Italian studies (with a related media impact) have been recently published. In particular, the study conducted by the University of Genoa found a protective effect of the ketogenic diet among COVID-19 patients [35]. Increasing the curiosity and interest of the general public, as well as the intermittent fasting diet, was a publication by Longo et al. [36]. Valter Longo is a well-known and internationally recognised scientist, who also has influence on the general public thanks to several educational publications he has edited. Moreover, during the COVID-19 pandemic, sedentary behaviours increased [19], which, even for short periods, negatively affected physical [37] and mental health [38]. The low level of physical activity, in association with isolation, also led to irregular eating patterns and frequent snacking [22,39], both of which are associated with increased caloric intake [40], increased risk of obesity [40], and higher prevalence of gastrointestinal symptoms [41]. The latter may have pushed people to seek out information about the FODMAP diet.

Our results could be extremely useful for science, practitioners, and policy makers for several reasons. Firstly, to the best of our knowledge, this is the first study assessing the internet users’ interest in diet by means of Big data analysis. This can open up new research questions and new applications of this method. Secondly, none of the previous studies assessed differences in seasonality, time period, and the COVID-19 pandemic. Third, from practitioners’ points of view, especially dieticians, these data are extremely useful because they can help determine in advance which of the (new) diets (particularly if considering those that are not evidence-based) are trendier among the general public. This helps practitioners to be better equipped during the counselling and to bring back the focus of the discussion on evidence-based healthy diets. Further, policy makers can also benefit from this analysis since these data offer an insight in the internet users’ behaviours. Indeed, it is extremely important to understand when, what and how to convey educational information, in order to promote more tailored internet educational interventions and save public money.

### Strengths and Limitations

Before generalising our results, some aspects should be taken into account. First of all, several different aspects may influence the Internet search peaks, as for instance news launched by mass media, scientific researchers’ results, or new rule introduction (such as, for instance, sugar taxation). It is important to consider that this type of data cannot be analysed on an exclusive basis. On the contrary, such data have to be considered complementary of traditional data collection systems. Moreover, even if the Internet has expanded and sped up the connectivity among countries, providing access to a large amount of data and information also to developing countries, not all people have the same level of access. In this perspective, this analysis is limited to people who have full or at least partial access to the web. Lastly, these types of analyses can be potentially affected by the so-called “filter bubble” effect, which was first introduced in 2011 and defined as the tailored results that the Internet search engines offer to users based on their preferences/previous searches [42].

On the contrary, these data contribute to the decrease of several biases, such as social desirability and recall bias, as well as representativeness of the sample. Moreover, the velocity of these data highly reduces the time lag between data collection and data analysis [11]. Lastly, Big Data and the novel data stream improve data dissemination [11].

## 5. Conclusions

In conclusion, the several data sources used in the current study confirm the high interest of the general public towards diet. Moreover, we showed that many different factors may influence internet users’ behaviours in searching information regarding diet—among them, seasonality, mediatic events, and, considering the current period, even the COVID-19 pandemic. Although the Mediterranean diet continues to represent the diet which raises the most interest, other diets (such as the ketogenic and macrobiotic diets) attracted the interest of the general population, with a seasonal cyclicity. In light of this, Big Data can offer new research opportunities providing timely data that might also be useful for policy makers and in terms of public health. In fact, these types of analyses could be useful to support information campaigns via the Internet, especially during the period when the population is more receptive.

## Figures and Tables

**Table 1:** Seasonal distribution and differences in the number of times a specific page was viewed by users.

Diets	Spring (Mean ± SD)	Summer (Mean ± SD)	Autumn (Mean ± SD)	Winter (Mean ± SD)	*p*-Value	*p*-Value Shapiro–Wilk Test
Mediterranean	20,950.3 ± 5229.7	9864.4 ± 4316.4	13,976.5 ± 7152.8	16,202.2 ± 4045.7	**<0.001**	0.989
Vegetarian diet (semi-vegetarian)	3413.5 ± 458.4	2733.8 ± 668.5	2852.4 ±721.3	3351.5 ±1007.5	**0.020**	0.417
Ketogenic diet	4304.2 ± 4121.8	3819.3 ± 3917.5	3295.7 ± 3135.2	3594.4 ± 3446.7	0.882	0.923
Atkins	839.5 ± 307.6	643.3 ± 249.9	527.4 ± 124.8	523.0 ± 237.6	**<0.001**	0.225
FODMAP	563.8 ± 777.5	605.4 ± 853.7	735.9 ± 877.3	666.6 ± 816.5	0.938	0.889
Acid–base diet	0.3 ± 0.6	1.0 ± 2.0	0.3 ± 0.6	0.4 ± 0.6	0.230	0.064
Vegan/Vegetalian	12.5 ± 6.8	9.2 ± 8.1	10.9 ± 10.5	9.5 ± 4.7	0.623	0.448
Blood groups diet	1150.6 ± 649.1	997.2 ± 606.5	1009.2 ± 714.3	1023.1 ± 585.7	0.901	0.051
Palaeolithic/Paleo diet	2077.5 ± 985.0	2118.7 ± 890.7	2180.1 ± 1089.1	1932.7 ±952.4	0.897	0.670
Scarsdale diet	664.5 ± 239.3	518.1 ± 128.9	424.1 ± 125.9	430.3 ± 209.6	**0.001**	0.254
Kousmine method	1357.6 ± 435.7	1235.3 ± 381.4	1240.5 ± 407.3	1209.0 ± 505.3	0.796	0.536
Zone Diet	2235.9 ± 621.5	1911 ± 744.8	1588.5 ± 497.2	1681.9 ± 735.6	**0.033**	0.632
Intermittent fasting	642.7 ± 1025.4	549.7 ± 798.9	386.3 ± 573.6	576.2 ± 980.4	0.842	0.521
Pescetarianism	4192.5 ± 1351.1	4048.9 ± 754.7	3413.3 ± 578.7	4650.8 ± 1991.7	**0.048**	0.874
Fruitarianism	3540.5 ± 1701.8	4092.1 ± 2379.5	3488.5 ± 1660.2	2987.7 ± 1418.9	0.381	0.799
Raw foodism	2900.3 ± 1252.3	2258.2 ± 713.6	2322.2 ± 832.2	2433.5 ± 902.4	0.220	0.184
Macrobiotics	5502.3 ± 5925.8	3431.7 ± 1127.1	3710.4 ± 1125.4	3502.7 ± 1141.4	0.174	0.051

SD: standard deviation; statistically significant (*p* < 0.05) differences have been reported in bold.

**Table 2:** Diet-related queries in the study period (2004–2021) using Google Trends.

Commonly Searched Diet-Related Queries	
Term in Italian (English Translation)	Search Volume (%)
Dukan dieta * (Dukan diet)	100
Dukan * (Dukan)	94
Dimagrire ^ (weight loss)	67
Dieta per dimagrire ^ (weight loss diet)	45
Dieta dimagrante ^ (slimming diet)	37
Dieta zona ^ (Zone diet)	34
Dieta Mediterranea (Mediterranean diet)	32
Dieta chetogenica/Dieta del gruppo sanguigno (Ketogenic diet/Blood group diet)	26
Dieta del riso */Dieta colesterolo */Dieta a zona ^/Dieta settimanale (Rice diet)/Cholesterol diet/Zone diet ^/Weekly diet	22
Colesterolo Cholesterol ^	21
Dieta Mozzi +/Diet plank (Mozzi’s diet/Plank diet)	18
Dieta vegetariana/Dieta proteica (Vegetarian diet/Proteic diet)	16
Dieta Lemme/Diete ° (Lemme’s diet/Diets)	15
Detox/Dieta gravidanza/Dieta vegana (Detox/Diet in pregnancy/Vegan diet)	14
Dieta ipocolarica/dieta detox (Low-calorie diet/detox diet)	13

* for this type of diet there is no corresponding Wikipedia page; ^ In Italian there are multiple ways to express the same concept; + The name refers to the promoter of the blood group diet; ° The name refers to the promoter of a high-protein diet.

## Data Availability

Data are freely downloadable from https://tools.wmflabs.org/pageviews/?project=en.wikipedia.org&platform=all-access&agent=user&range=latest-20&pages= (accessed on 22 March 2021).

## Supplementary Materials

The following are available online at https://www.mdpi.com/article/10.3390/nu13113683/s1, Figure S1: Google Trends data from 1 January 2004 to 26 March 2021 for the research trends of the word “dieta” (diet in English) in Italy. Figure S2: Wikipedia curves with reference to the mean number of page views by year for Mediterranean, vegetarian (semi-vegetarian), ketogenic, Atkins, FODMAP, acid–base, vegan, blood group, palaeolithic, Scarsdale, Kousmine method, zone diet, intermittent fasting, pescetarianism, fruitarism, raw food, and macrobiotics diets. Figure S3: (a) Google Trends data from 1 January 2004 to 26 March 2021 for the research trends of the word “Mario Pianesi” in Italy; (b) geographical distribution of the search volume. Figure S4: Number of articles indexed in PubMed by year, for ketogenic, Mediterranean and macrobiotic diets. Table S1. Definitions of the diets analysed, and differences in the number of times a specific page was viewed by users before and during the COVID-19 pandemic.
