# Supplementary material for: Wikipedia, Google Trends and Diet: Assessment of Temporal Trends in the Internet Users’ Searches in Italy before and during COVID-19 Pandemic
Source: Nutrients. 2021 Oct 20;13(11):3683. doi: 10.3390/nu13113683 (PMC8620684; doi:10.3390/nu13113683)
Supplement: Supplementary file 1 [file nutrients-13-03683-s001.zip › nutrients-1411202-supplementary.pdf]

Supplementary Figure S1. Google Trends data from 1 January 2004 to 26 March 2021 for the research trends of the word “dieta” (diet in English) in Italy.

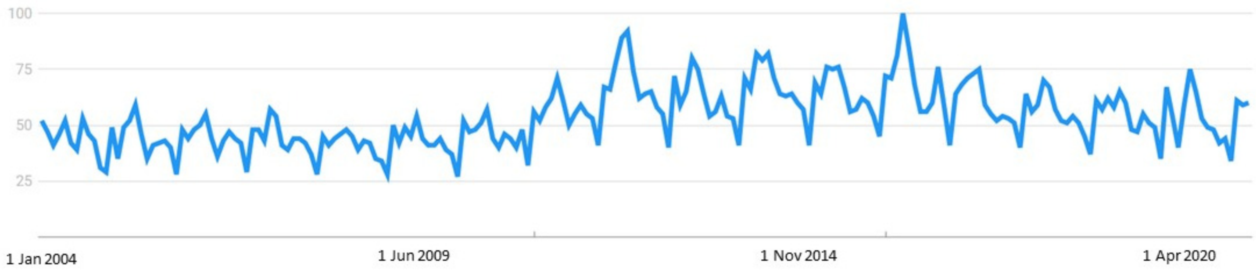

Supplementary Figure S2 Wikipedia curves with reference to the mean number of page views by year for Mediterranean, Vegetarian (semi-vegetarian), Ketogenic, Atkins, FODMAP, Acid-base, Vegan, Blood group, Paleolithic, Scarsdale, Kousmine method, Zone diet, Intermittent fasting, Pescetarianism, Fruitarianism, Raw foodism, Macrobiotics diets.

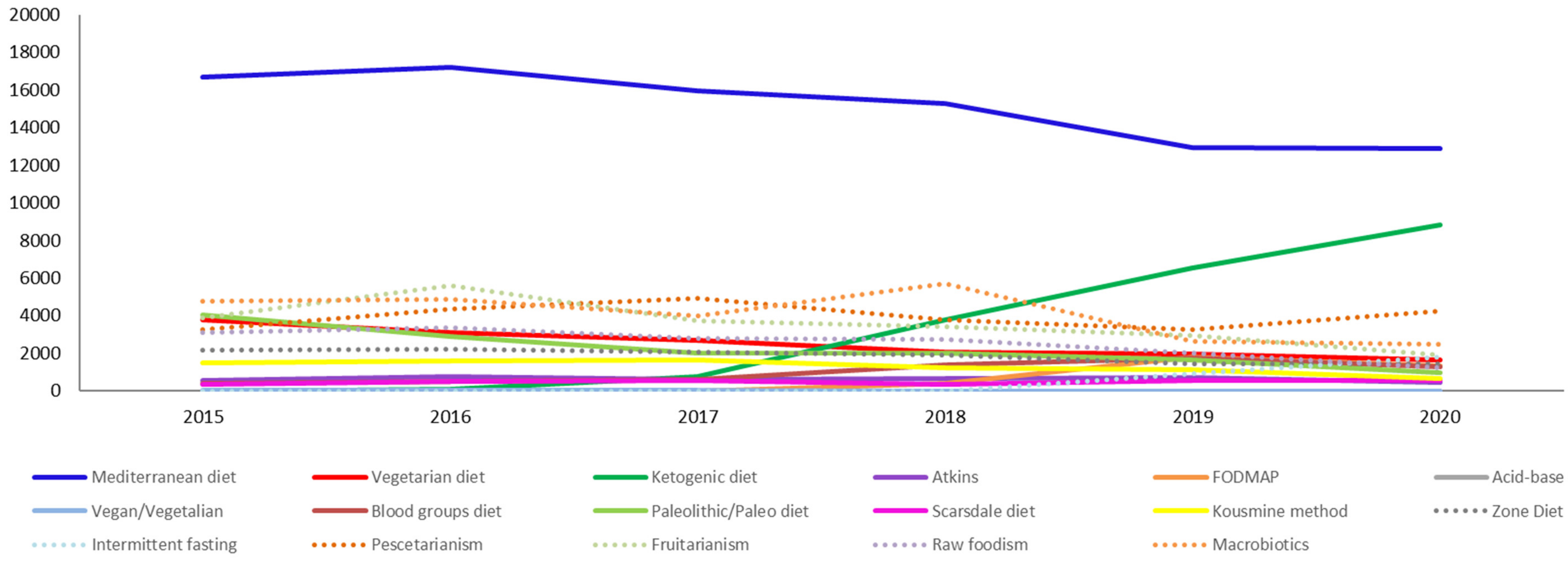

Supplementary Figure S3. a) Google Trends data from 1 January 2004 to 26 March 2021 for the research trends of the word “Mario Pianesi” in Italy; **b)** geographical distribution of the search volume.

a)

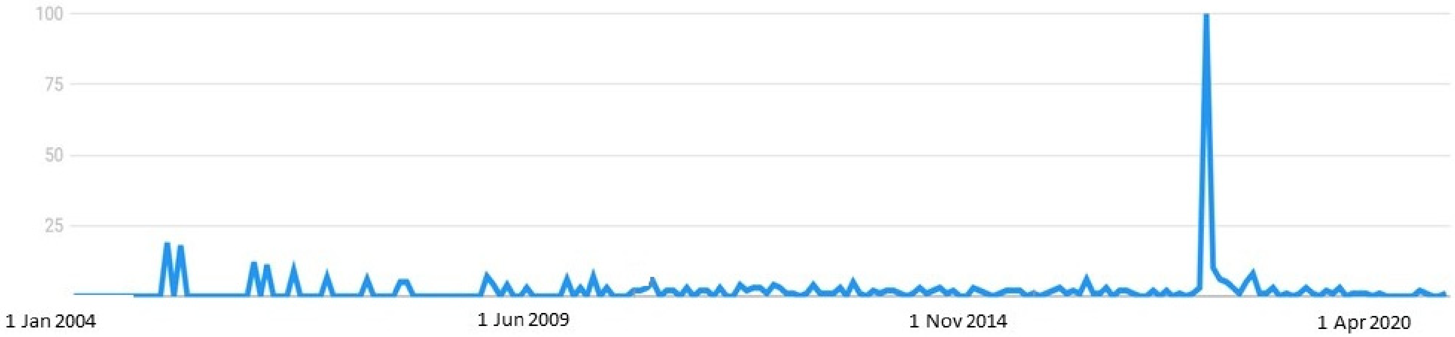

b)

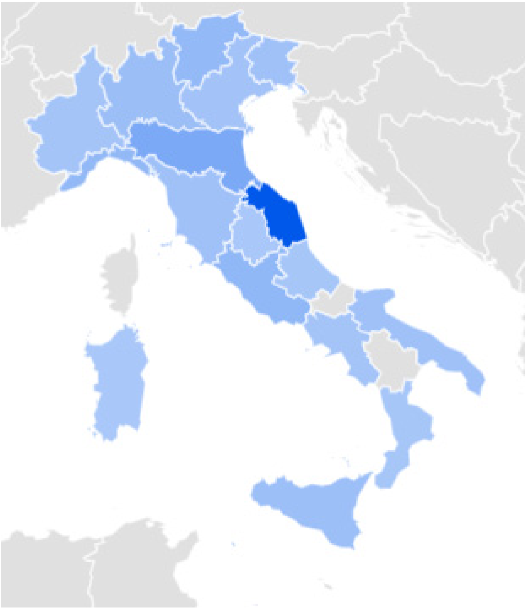

Supplementary Figure S4. Number of articles indexed in PubMed by year, for Ketogenic, Mediterranean and Macrobiotic diets.

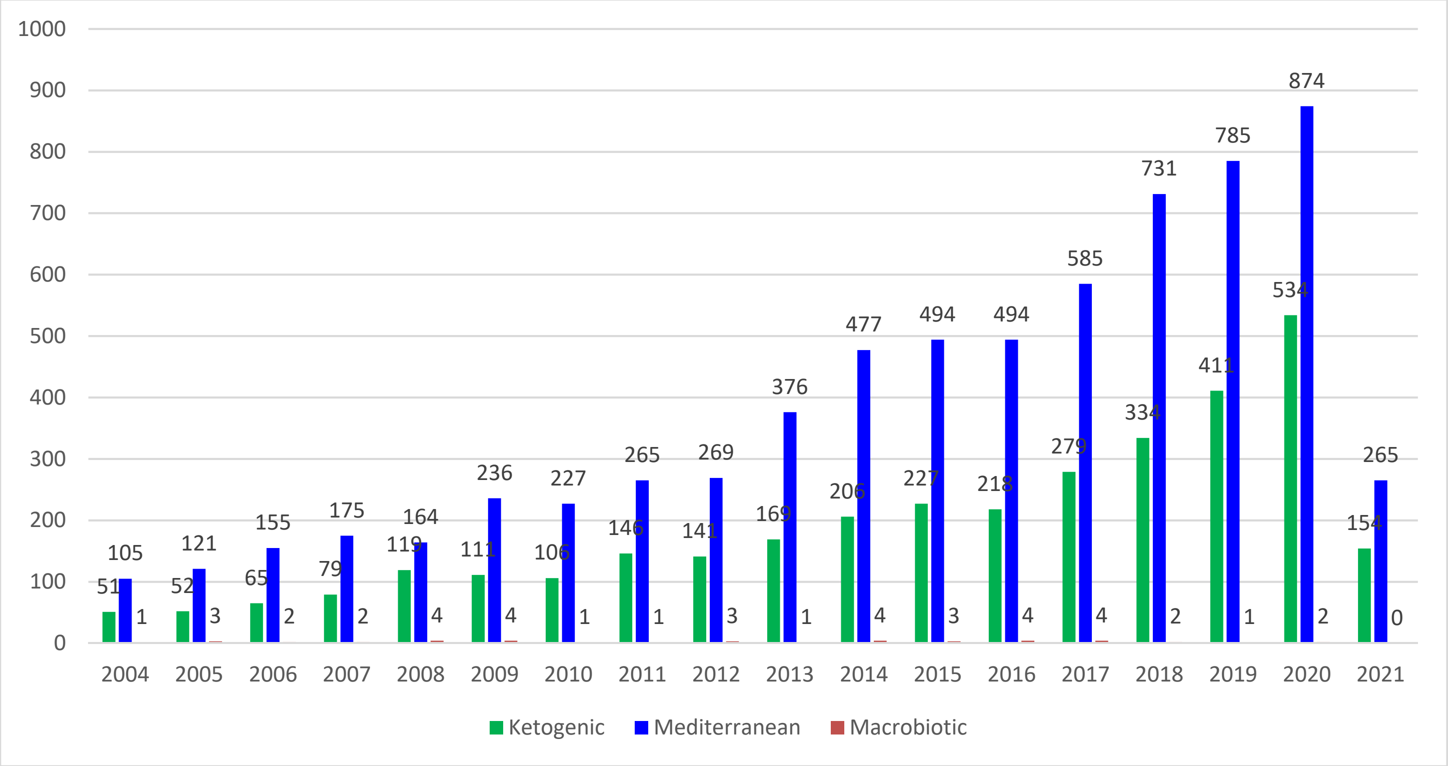

Supplementary Table S1. Definitions of the diets analyzed, and differences in the number of times a specific page was viewed by users before and during the COVID-19 pandemic.

Diets	Definition of diets, according to Wikipedia	Before COVID-19 (mean ± SD)	During COVID-19 (mean ± SD)	p-value
Mediterranean	Diet with high consumption of olive oil, legumes, unrefined cereals, nuts, fruits, and vegetables, moderate to high consumption of fish, moderate consumption of dairy products and wine, and low consumption of other animal products and refined foods.	15,606.4 ± 6667.3	12,273 ± 5368.9	0.1239
Vegetarian diet (semi-vegetarian)	Diet exclusively based on plants origin foods, without meat (but including eggs, milk and dairy).	3174.2 ± 790.8	2567.2 ± 573.3	**0.0185**
Ketogenic diet	Diet high in fat, with low-moderate protein, and low carbohydrates	2717.1 ± 2888.9	8887.2 ± 2008.5	**<0.0001**
Atkins	Low-carbohydrate, high-fat, high-protein diet.	658.8 ± 267.3	456.3 ±354.2	**0.0180**
FODMAP	Diet with low fermentable carbohydrates recommended for managing patients with irritable bowel syndrome.	525.2 ± 826.3	1265.1 ± 998.7	**0.0052**
Acid-base diet	Diet based on the misconception that different types of food can have an effect on the body pH balance.	0.6 ± 1.2	0.1 ± 0.3	0.1896
Vegan / Vegetalian	Diet exclusively based on plants origin foods, without any type of animal products.	11.5 ± 8.0	5.2 ± 3.6	**0.0145**
Blood groups diet	Diet defined according to the blood group system.	1015.4 ± 674.5	1173.4 ± 304.8	0.4511
Paleolithic / Paleo diet	Diet based on roots, meat, nuts, vegetables, fruits, low processed food and no dairy products, grains, sugar, legumes, processed oils, salt, alcohol, and coffee.	2302.3 ± 897.3	940.9 ± 845.8	<0.0001
Scarsdale diet	Low-carbohydrate, high-protein diet with restrictions in some plant-origin foods.	482.5 ± 172.8	609.7 ± 290.9	0.0528
Kousmine method	Diet based on fruits, vegetables, vitamins supplements and whole grains but with uncooked grain and seed, and no saturated fats.	1395.9 ± 334.5	598.5 ± 90.9	**<0.0001**

Zone Diet	Low-carbohydrate diet intended to maintain insulin sensitivity, avoiding insulin relies spikes. Weight reduction is obtained due to calorie restriction.	1940.5 ± 664.3	1322.2 ± 579.1	**0.0054**
Intermittent fasting	Diet based on calorie restriction obtained whit alternate-day fasting.	266.2 ± 572.4	1893.4 ± 1460.7	**<0.0001**
Pescetarianism	Plant-based diet with seafoods as the only source of meat.	3941.8 ± 1339.7	4680.2 ± 1175.8	0.0936
Fruitarianism	Diet with only fruits, nuts and seeds.	3833.3 ± 1832.7	1962.9 ± 609.0	**0.0014**
Raw foodism	Diet based only on raw foods.	2728.4 ± 788.8	1115.5 ± 268.4	**<0.0001**
Macrobiotics	Diet derived by Zen Buddhism, based on a balance between yin (negative) and yang (positive) foods meaning a local and seasonal foods consumption, low animal products intake, and a moderate meal consumption.	4294.9 ± 3208.4	2426.5 ± 333.7	0.0595
